# Differences in Muscle Activation Patterns during Sit to Stand Task among Subjects with and without Intellectual Disability

**DOI:** 10.1155/2013/173148

**Published:** 2013-10-07

**Authors:** Antonio I. Cuesta-Vargas, Manuel González-Sánchez

**Affiliations:** ^1^School of Clinical Sciences, Faculty of Health, Queensland University of Technology (QUT), Victoria Park Road, Kelvin Grove, QLD 4059, Australia; ^2^Department of Physiotherapy, Faculty of Health Sciences, University of Malaga, 29071 Málaga, Spain

## Abstract

The aim of this study is to analyse the differences in muscle activity between subjects who have intellectual disability and healthy subjects when they make the transition from sitting to standing positions. A cross-sectional study. A group of adults was divided into two subgroups: with and without intellectual disability (ID). The means of the basic features in both groups were 22.13 and 22.83 for age, 66.38 and 67.67 for weight, and 173.38 and 174.33 for height, for the ID (*n* = 8) and without ID (*n* = 7) groups, respectively. Each subject performed three sets of five repetitions during which, starting from sitting, they had to get up and sit on the chair. The recording of muscle activity was performed using surface electromyography taking the measures of muscle activity of different muscles of the lower limbs. The results showed differences in the pattern of muscle activity between groups during sitting to standing movement.

## 1. Introduction

Muscle activation (MA) that generates force is required for many activities of daily living (ADL), including eating, dressing, walking, or rising from a chair (sit to stand movement (STS)). Each of these activities is essential to ensure the maintenance of a person's physical independence [1]. Therefore, it is not surprising to discover that the progressive loss of effectiveness in MA is associated with a deficiency of basic capabilities, such as those previously mentioned and which, therefore, decreases a person's independence [2].

The sequences of STS and sitting are very common in ADL. The ability to develop STS movements is considered a very important feature for determining the degree of independence and the quality of life of a person [2]. This sequence is considered the most mechanical movement within ADL [3, 4], due to the high level of muscle activation that it requires, as individuals need to coordinate a transfer from a horizontal to a vertical position in one movement [5]. Furthermore, it is necessary for nervous system stability and alignment of the various body segments both dynamically [6] and statically. Nevertheless, for the realisation of this sequence, muscle strength [7, 8], balance [9, 10], and synergy of muscle activation [11, 12] are required.

Individuals with intellectual disabilities (ID) often have different MAs from those who do not have ID [13–15]. It appears that this MA would result in a poorer physical profile whose explanations could be found in chronotropic failure, reduced motivation, or even in the difficulty of advising this population [16]. It appears that this difference in the MA is associated with qualitative and quantitative deficiencies of muscle tissue, especially related to a sedentary lifestyle [14]. On the other hand, fitness in people with ID appears to be closely related to the vocational performance level of the individual and it has been shown that when individuals were removed from competitive environments, there was a decline in the quality of life and ability to perform the tasks [17, 18], leading to a progressive increase in the dependency of the person and a decline of their integration in society [1, 19].

MA could be defined as the distribution of activation or muscle strength of individual muscles to produce a joint motion [20], which can be studied using electromyography (EMG), using different models to estimate muscle strength [21]. In addition, several studies have shown that a muscle may act as tonic or phasic muscle depending on the subject's posture or movement performed [22, 23].

In studies that have examined muscle coordination, surface electromyography (sEMG) has been used more frequently than intramuscular electromyography (imEMG) for different reasons: the imEMG is invasive, requires medical personnel, can cause tissue damage, and can cause pain in muscles during muscular contraction, which limits the number of muscles that can be studied simultaneously. In addition, the volume of muscle that can be measured is reduced to a few cubic millimetres, which cannot represent the entire muscle mass involved during movement [24].

MA is therefore critical, whether in people with or without disabilities, to move in an effective and coordinated way, which is considered ineffective in chronic cases of ID [25]. In particular in the lower limbs, this capability ensures greater autonomy in all activities performed during daily life, whether professional or personal [26, 27]. Furthermore, it has been observed that paraspinal muscles and abdominal muscles are essential for posture control, in either static or dynamic situations [28, 29].

Due to the increasing number of people with ID in society and the progressive reduction in mortality due to the evolution of science [27], the maintenance and development of muscle strength are important aspects of the person to favour the possibility of performing activities over a longer period of time [30].

The clear relationship between good physical health and longevity in individuals with ID [31] makes the need to identify the way that the neuromuscular ability is distributed to generate force in the ADLs of people with ID critical, so that more focused intervention strategies can be created to compensate for any differences between a healthy person and one with an intellectual disability.

The STS movement has been studied using different instruments [1, 32, 33] on different population groups [1, 32–35], although no studies were found that used electromyography as a tool to analyse the pattern of behaviour of people with ID at the time of performing the task in comparison with STS subjects without ID. However, to analyse the STS sequence execution comparing healthy people with ID is important because it could provide opportunities for intervention to maintain or improve the performance of this sequence in people with ID, through a rehabilitation program or as a part of a supervised exercise planning, contributing to their independence in activities of daily living.

The objective set out in this study is to analyse the differences in MA between subjects who have ID and healthy subjects when they make the transition from sitting to standing positions by placing the point (sitting) at two different heights and trying to define patterns of movement between both groups. The study hypothesis is that there will be significant differences between the groups when assessing MA.

## 2. Materials and Method

### 2.1. Participants

A cross-sectional study was performed in which a group of adults was divided into two subgroups: with or without intellectual disability (mild/moderate). All participants must be between 18 and 40 years old. Besides, the BMI should not be higher than 30 (Kg/m^2^). In addition, all subjects of the ID group must be administratively defined as subjects with mild (IQ 70–50) to moderate (IQ 50–35) ID after administered test (psychological and educational) by the integration program for people with ID “Special Olympics.”

Exclusion criteria used were as follows: participants with severe ID, coexisting orthopaedic impairments, cardiovascular or pathologies, or a BMI over 30 (Kg/m^2^) or who were pregnant were excluded. 

All participants gave informed consent voluntarily, following the guidelines of the Helsinki Declaration of 1964, which sets out ethical principles for all inquiries with humans and has been upgraded in successive meetings of the World Medical Association (Declaration of Helsinki 2009 [36]). In addition, this study was approved by the University of Malaga Ethics Committee.

### 2.2. Material

#### 2.2.1. Electromyography

The recording of muscle activity was performed using surface electromyography (MegaWin 3.0, Mega Electronics Ltd, Kuopio, Finland), taking the measures of MA of the following muscles: vastusmedialis quadriceps (QVM), rectus femoris of the quadriceps (QRF), long head of biceps femoris (BF), tibialis anterior (TA), medial gastrocnemius (MGN), rectus abdominus (RA), erector spinae (ES) and soleus (S). 

**Figure 1:**
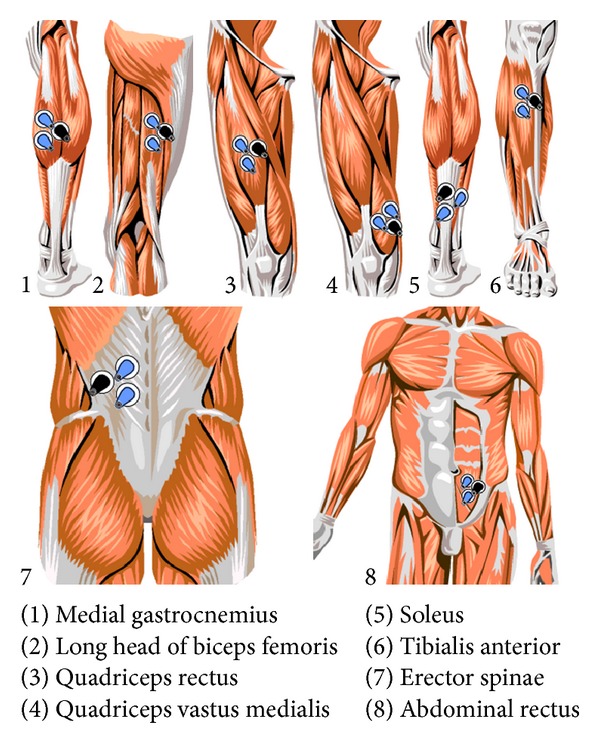
Schematic placement of surface electrodes on each muscle. From MegaWin 3.0.

On each muscle three electrodes were placed using a circular adhesive Ag-AgCl in the right hemisphere of each participant at a distance of two inches between them. To encourage the proper adhesion of the electrodes to each participant, body hair was removed using an electric razor, and to minimise skin resistance the area was washed with alcohol [37] (SENIAM).  Figure 1 shows a schematic of the positioning of each of the electrodes in the different muscles studied.

Each participant started the movement after receiving the indication of the investigator. Before the command, 5-seconds of data were collected in each test.

#### 2.2.2. Digital Metronome

To ensure the execution of the sequence STS at the same speed, a digital metronome (Qwik Time QT-5 Metronome, China) was used.

### 2.3. Methods: Procedure

All of the measurements were performed by the same investigator, while an assistant was always present to increase the reliability of the evidence. All participants were provided with the same instructions and directions and all tests were performed under the same conditions.

Each participant had the possibility of an initial test of the movement that was being investigated, so that they could become familiar with the sequence executed correctly as well as the necessary speed, which was indicated by the digital metronome. During this test, the participants received feedback from researchers on how to perform the STS sequence. Subjects were instructed to keep their head facing forward, flex their hips forward, transfer their weight forward, and then begin the act of getting up in order to end up in the final position with their hips and knees extended [38].

Each subject performed three sets of five repetitions during which, starting from sitting, they had to get up and sit on the chair at a rate of 10 times per minute. This speed was indicated by a metronome sound at the foot of the chair. This sequence was performed from two different chair heights: 43 and 38 centimetres high.

The participants were seated in chairs without arms and without supports, with their arms being folded across their chest, resting their hands on the opposite shoulder with their feet being open to the hips. Participants had to get up, extend their knees and trunk, and return to a seated position at the speed set by the metronome sound. The researchers made sure that the subjects fully extend their trunk and knees before they began the movement to return to sitting. The values considered for statistical analysis were the means of three recordings made at two different heights. If some repetitions were poorly executed, the researchers stopped the trial and began again.

#### 2.3.1. Data Processing and Reduction

The raw electromyographic signal was passed through 12-bit analogue to digital converter through a sampling frequency of 1000 Hz. This was subsequently passed to a computer for further analysis. Background noise in the filtered signal was lower than the gross and the electromyographic signal was filtered using low- and high-pass filters (Butterworth) with a bandwidth between 20 and 500 Hz. Each of the records taken in each muscle was normalised on the basis of calculating the total gesture, in order to produce a pattern of expression in terms of the participation of each muscle.

### 2.4. Statistical Analysis

A descriptive study of the participants was performed, and then the relative contribution of each muscle in the sequence was estimated. The data collected were analysed using version 19.0 of the SPSS statistical software.

## 3. Results

15 volunteers participated in this study, who were divided into two groups based on whether or not they had intellectual disability. ID means intellectual disability (*n* = 8) and WID means without intellectual disability (*n* = 7). The means of the basic features in both groups were 22.13 and 22.83 for age, 66.38 and 67.67 for weight, and 173.38 and 174.33 for height, for the ID and WID groups, respectively.

**Figure 2:**
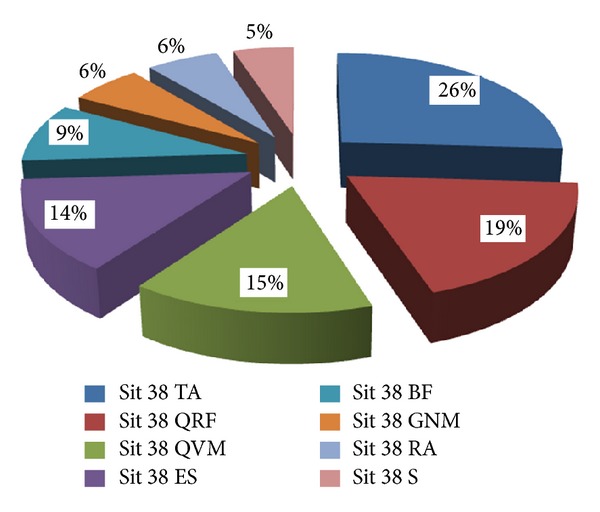
Distribution of muscle activation in ID subjects. 38 cm.

**Figure 3:**
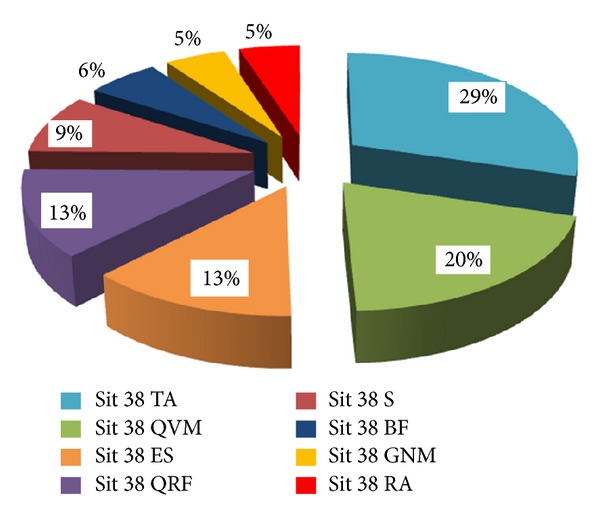
Distribution of muscle activation in WID subjects. 38 cm.

Table 1 shows the mean normalised electromyographic recordings based on each muscle involvement in the embodiment of the seat sequence when STS was at a height of 38 cm. Thus, Figures 2 and 3 provide a more complete overall sequence, showing the relative share of each muscle when the subject is in a seat that is 38 cm high.

**Figure 4:**
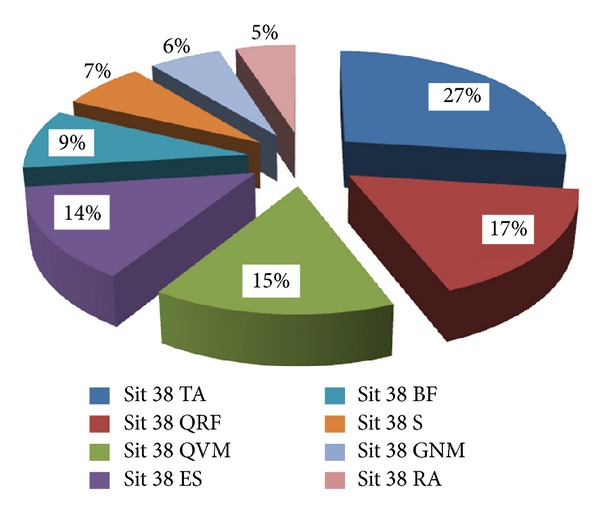
Distribution of muscle activation in ID subjects. 43 cm.

**Figure 5:**
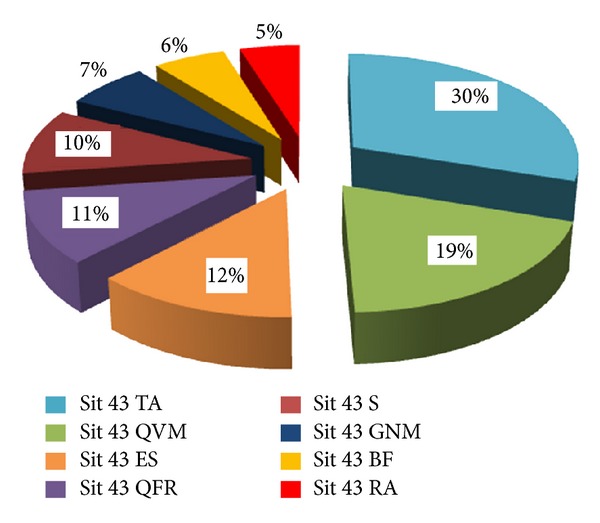
Distribution of muscle activation in WID subjects. 43 cm.

**Figure 6:**
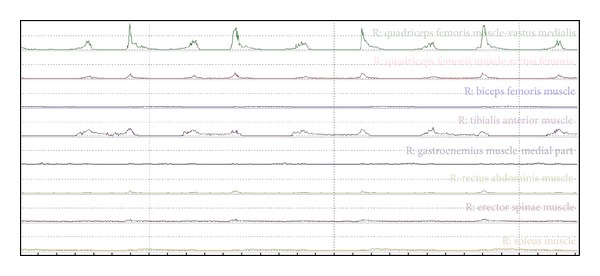
Example of an electromyographic recording with the activation of each muscle.

In addition, Table 2 shows the normalised distribution of the entire STS movement when the seat was at a height of 43 cm. Figures 4 and 5 allow more global results to be seen and indicate how three of the muscles have a stake in the sequence that is far more important than the sum of all of the others. An example of an electromyographic recording can be seen in Figure 6.

## 4. Discussion

The aim of this study was to analyse the differences in MA between people who have an ID and healthy subjects when transitioning from sitting to standing. The study also involved taking two different heights of chairs as a starting point. The hypothesis of this study leaned towards the existence of differences between the groups at the time of executing the analysed sequence. After conducting the study it could be seen that analysis of the data could partially confirm the hypothesis.

To our knowledge, this is the first study that has recorded MA through electromyography for a sequence that is very important to daily living, such as getting up and sitting on a chair, in healthy subjects compared with people with ID.

When analysing the results, it can be seen that subjects with ID seem to have a different neuromuscular response from subjects WID. However, three muscles (TA-QVM-QRF in subjects with ID, and TA-QVM-ES for subjects WID) appeared to contribute more than half of the MA necessary to perform the STS task. Specifically, in the case of individuals with ID the sum of the three muscles was 60% and 69%, and in the WID group this was 62% and 61% when performing the STS movement from 38 to 43 cm, respectively.

The neuromuscular recruitment appears to hold for each group. Furthermore, in the neuromuscular activity measurements, the variance (SD) is very low. Therefore, if the relative recruitment of MA and the variance of the measurements of each muscle are combined, a particular movement pattern can be seen within each group. In addition, a previous study suggests that the change in seat height can cause a change in the implementation strategy of a movement [39].

Other authors have analysed this action in response to a series of measures, such as lumbopelvic angle [40], kinematic and kinetic parameters [41], or the angular acceleration [42]. Specifically, the latter study reports the change of strategy and therefore a distribution of MA, causing people who have muscle weakness to increase the load (e.g., through body weight) when they perform the STS task.

In the literature, two ways to perform and define the strategy for implementing the STS task were found. Defined as the “momentum-transfers strategy,” the first method implies that the subject in question makes a small trunk flexion of the weight transferring forwards and then begins the separation of the seat, ending with the starting foot. This form is most common among healthy people [38, 40, 41]. Another way to make the sequence is increasing trunk flexion before starting to move from the chair [42], which is usually performed by people with muscle weakness in the legs.

If the results obtained in this study are analysed, in both groups there are four muscles that act primarily as stabilisers of motion (TA-RA-LMC-S) and four others that are responsible for implementing the sequence (QRF-QVM-EN-BF). The main difference in the pattern of movement when performing the STS task lies precisely in this last group of muscles. In the group of people with ID, a greater relative activation of the QRF and the QVM with respect to the ES and BF is observed. However, in the group of WID subjects, it is seen how this activation is further offset relative to the third muscle being the ES muscle, with which neuromuscular activation is concerned. This seems to imply that individuals with ID have a pattern of movement in which the torso flexes very little, so they need greater quadriceps activation and, consequently, rise in a much more vertical manner than the WID group. The WID group have patterns that are more similar to those observed in previous studies [38, 40, 42], subjects without intellectual disabilities apparently prepare the first step along the execution of the sit to stand movement, while people with ID need to first stabilise their body before they start walking in a second sequence.

The explanation for this difference could be found in the conclusions reached by Skowroński et al. (2009) [39] who asserted that the physical test results may be influenced by gender, age, and also ID. On the other hand, other studies have shown that people who have ID have a lower balance, both static and dynamic, than those without ID [40–43], which may explain the difference in MA as the difficulty in implementing STS must be considered as they will need to compensate for a lack of balance.

Studies have shown that supervised training in subjects with ID improves physical performance in sports and in ADLs [44]. Thus, appropriate physical activity [43] should favour the health of individuals with ID and have a diminished impact on those basic features shown, such as balance [40, 41], resistance, or strength [14, 45].

The main strength of this study is that this is the first study to compare the MA of the major muscles of the lower limb and trunk involved in the sequence of STS, in order to identify a pattern of movement in both groups. This discovery may help to optimise the training sessions for those individuals who have ID. This finding could encourage each person to improve, depending on the case, their ability in ADL or sports performance.

A weakness of this study is that the population of people with ID is limited to those with a mild or moderate level of disability, so it would be necessary in the future to study groups with different levels of ID (mild-moderate-severe-very severe) and compare them with people without ID. This would determine how muscle recruitment changes with the level of involvement of ID. Moreover, the results obtained they are may be determined by the degree to which participants feel part of a study and, therefore, change their normal patterns of movement.

## 5. Conclusion 

The main conclusion is that there is a difference in the pattern of muscle activation between participants with and without ID during STS. This difference is observed as the muscle with the greatest activation as QVM for the WID and the TA group and QRF for the group with ID within the STS pattern. 

## Figures and Tables

**Table 1:** Mean distribution of the muscle leg activation during stand up and sit down from a chair with a seat pan height of 38 cm.

ID		WID	
Sit 38 TA	0.26	Sit 38 TA	0.30
SD	±0.06	SD	±0.06
Sit 38 QRF	0.19	Sit 38 QVM	0.20
SD	±0.06	SD	±0.09
Sit 38 QVM	0.15	Sit 38 ES	0.13
SD	±0.03	SD	±0.05
Sit 38 ES	0.14	Sit 38 QRF	0.13
SD	±0.04	SD	±0.05
Sit 38 BF	0.09	Sit 38 S	0.09
SD	±0.02	SD	±0.01
Sit 38 GNM	0.06	Sit 38 BF	0.06
SD	±0.01	SD	±0.03
Sit 38 RA	0.06	Sit 38 GNM	0.05
SD	±0.03	SD	±0.01
Sit 38 S	0.05	Sit 38 RA	0.05
SD	±0.02	SD	±0.01
*N*	8		7

Note: QVM: quadriceps vastusmedialis; QRF: quadriceps rectus femoris; BF: biceps femoris; TA: tibialis anterior; GNM: gastrocnemius medialis; RA: rectus abdominis; ES: erector spinae; S: soleus; ID: intellectual disability group; WID: without intellectual disability group.

**Table 2:** Mean distribution of the muscle leg activation during stand up and sit down from a chair with a seat pan height of 43 cm.

ID		WID	
Sit 43 TA	0.27	Sit 43 TA	0.31
SD	±0.06	SD	±0.08
Sit 43 QRF	0.17	Sit 43 QVM	0.20
SD	±0.06	SD	±0.09
Sit 43 QVM	0.15	Sit 43 ES	0.13
SD	±0.03	SD	±0.04
Sit 43 ES	0.14	Sit 43 QFR	0.11
SD	±0.06	SD	±0.06
Sit 43 BF	0.09	Sit 43 S	0.10
SD	±0.02	SD	±0.02
Sit 43 S	0.07	Sit 43 GNM	0.07
SD	±0.03	SD	±0.04
Sit 43 GNM	0.06	Sit 43 BF	0.06
SD	±0.02	SD	±0.02
Sit 43 RA	0.05	Sit 43 RA	0.05
SD	±0.01	SD	±0.01
*N*	8		7

Note: QVM: quadriceps vastusmedialis; QRF: quadriceps rectus femoris; BF: biceps femoris; TA: tibialis anterior; GNM: gastrocnemius medialis; RA: rectus abdominis; ES: erector spinae; S: soleus; ID: intellectual disability group; WID: without intellectual disability group.
